# RNA-sequencing investigation identifies an effective risk score generated by three novel lncRNAs for the survival of papillary thyroid cancer patients

**DOI:** 10.18632/oncotarget.18274

**Published:** 2017-05-26

**Authors:** Yi-Huan Luo, Liang Liang, Rong-Quan He, Dong-Yue Wen, Guo-Fei Deng, Hong Yang, Yun He, Wei Ma, Xiao-Yong Cai, Jun-Qiang Chen, Gang Chen

**Affiliations:** ^1^ Department of Gastrointestinal Surgery, First Affiliated Hospital of Guangxi Medical University, Nanning, Guangxi Zhuang Autonomous Region, P.R. China; ^2^ Department of General Surgery, First Affiliated Hospital of Guangxi Medical University (West Branch), Nanning, Guangxi Zhuang Autonomous Region, P.R. China; ^3^ Department of Medical Oncology, First Affiliated Hospital of Guangxi Medical University, Nanning, Guangxi Zhuang Autonomous Region, P.R. China; ^4^ Department of Ultrasonography, First Affiliated Hospital of Guangxi Medical University, Nanning, Guangxi Zhuang Autonomous Region, P.R. China; ^5^ Department of Pathology, First Affiliated Hospital of Guangxi Medical University, Nanning, Guangxi Zhuang Autonomous Region, P.R. China

**Keywords:** papillary thyroid cancer, lncRNA, prognosis, AC079630.2, CRNDE

## Abstract

Scholars are striving to apply molecular biology involving long non-coding RNA (lncRNA) in the prognostication of papillary thyroid cancer (PTC). However, the clinical role of lncRNAs in the prognostic setting of PTC is still unclear. Herein, a comprehensive inquiry was performed to screen lncRNA expression profiling with 507 PTC patients from The Cancer Genome Atlas RNA-sequencing datasets. A total of 734 lncRNAs were detected to be aberrantly expressed, among which three novel lncRNAs including AC079630.2, CRNDE and CTD-2171N6.1 were markedly related to the progression and survival of PTC. Furthermore, the aberrant expression of these lncRNAs could be verified by other cohorts from gene expression omnibus (GEO) as detected by microarrays. Next, we established a three-lncRNA signature and divided the PTC patients into two subgroups of high- and low-risk. Interestingly, patients with high-risk tended to gain obviously poorer outcome. Most importantly, this three-lncRNA signature was an independent biomarker to predict the patient survival of PTC. The accurate molecular roles of these three lncRNAs remains unclarified and warrants further characterization, but our current data propose that they might play pivotal roles in PTC tumorigenesis and more importantly, these novel lncRNAs are closely related to patients’ survival. These discoveries will have far-reaching consequences with respect to molecular prediction of PTC.

## INTRODUCTION

Thyroid cancer is the most widespread class of endocrine malignancies. The occurrence of thyroid cancer has been gradually growing over the last 30 years [1, 2]. It was estimated that 64,300 new cases of thyroid cancer occurred in the United States in 2016, and 1,980 of them deceased (https://www.cancer.org/). Women are more prone to be affected by thyroid cancer than men. People between the ages of 25 and 65 are the high-risk group to suffer thyroid cancer [3, 4]. There are four subtypes of thyroid cancer according to the histopathological examination, including papillary, follicular, medullary and anaplastic thyroid cancer. Generally, thyroid cancer is divided into two classes: well-differentiated (papillary or follicular) and poorly-differentiated (medullary or anaplastic), which also lead to various clinical outcomes. For most of thyroid cancer, the therapeutic options including operation, radioactive iodine ablation, and thyroid stimulating hormone (TSH) suppressive therapy provide an overall survival (OS) rate of 97.7% at 5 years. Nonetheless, locoregional recurrence takes place in up to 20% of thyroid cancer patients. More seriously, distant metastases will occur in almost 10% of thyroid cancer patients in 10 years [5–7].

The molecular biology has been greatly improved in the prognostication of papillary thyroid cancer (PTC). Several molecular biomarkers have been commended for the risk stratification for PTC, along with traditional clinicopathological parameters. The typical examples are BRAF^V600E^ and telomerase reverse transcriptase (TERT) [8–14]. Nonetheless, the clinical characteristics of molecular characterization in the prognostic setting of PTC still remain unclarified. A deeper understanding of the drivers and molecular mechanisms behind PTC tumorigenesis, as well as documentation of candidate prognostic markers, is essential to put forward better diagnostic and therapeutic strategies for PTC [15–17].

Long noncoding RNAs (lncRNAs) have been proved to play essential roles in cancer biology, including PTC. The detection of transcriptome profile of PTC is an ideal option to screen and discover new lncRNA candidates [18]. By far, several groups performed genome-wide investigation into lncRNAs expression profile in PTC using microarray [19, 20] or RNA sequencing [21–23]. However, these profilings were all based on a small number of cases and the results were varying with each other. The cancer genome atlas (TCGA) has made great efforts to unveil the molecular events of various cancers, including the role of lncRNAs. Ma et al. [24] mined the data from TCGA dataset and they reported 220 lncRNAs with altered expression from the annotated 2773 lncRNAs which were approved by the HUGO gene nomenclature committee via cBioPortal. They further reported that FAM41C, CTBP1-AS2, LINC00271, HAR1A, LINC00310 and HAS2-AS1 were related to recurrence of PTC patients. More importantly, only one lncRNA, LINC00271 was identified to be an independent risk factor for the progress and recurrence of PTC in multivariate analyses. In the current study, the genome-wide lncRNA expression data from the high-throughput RNA-sequencing dataset was also achieved from TCGA; however, 7589 lncRNAs were annotated by Ensembl genome browser (http://www.ensembl.org/). More interestingly, we used DEseq R package to screen the differentially expressed lncRNAs and the results differed from Ma et al. [24]. We further selected those significant prognostic lncRNAs to form a signature. The three-lncRNA signature can be an effective independent indicator to predict the progression and survival of PTC.

## RESULTS

### Selection of differentially expressed lncRNAs

**Figure 1:**
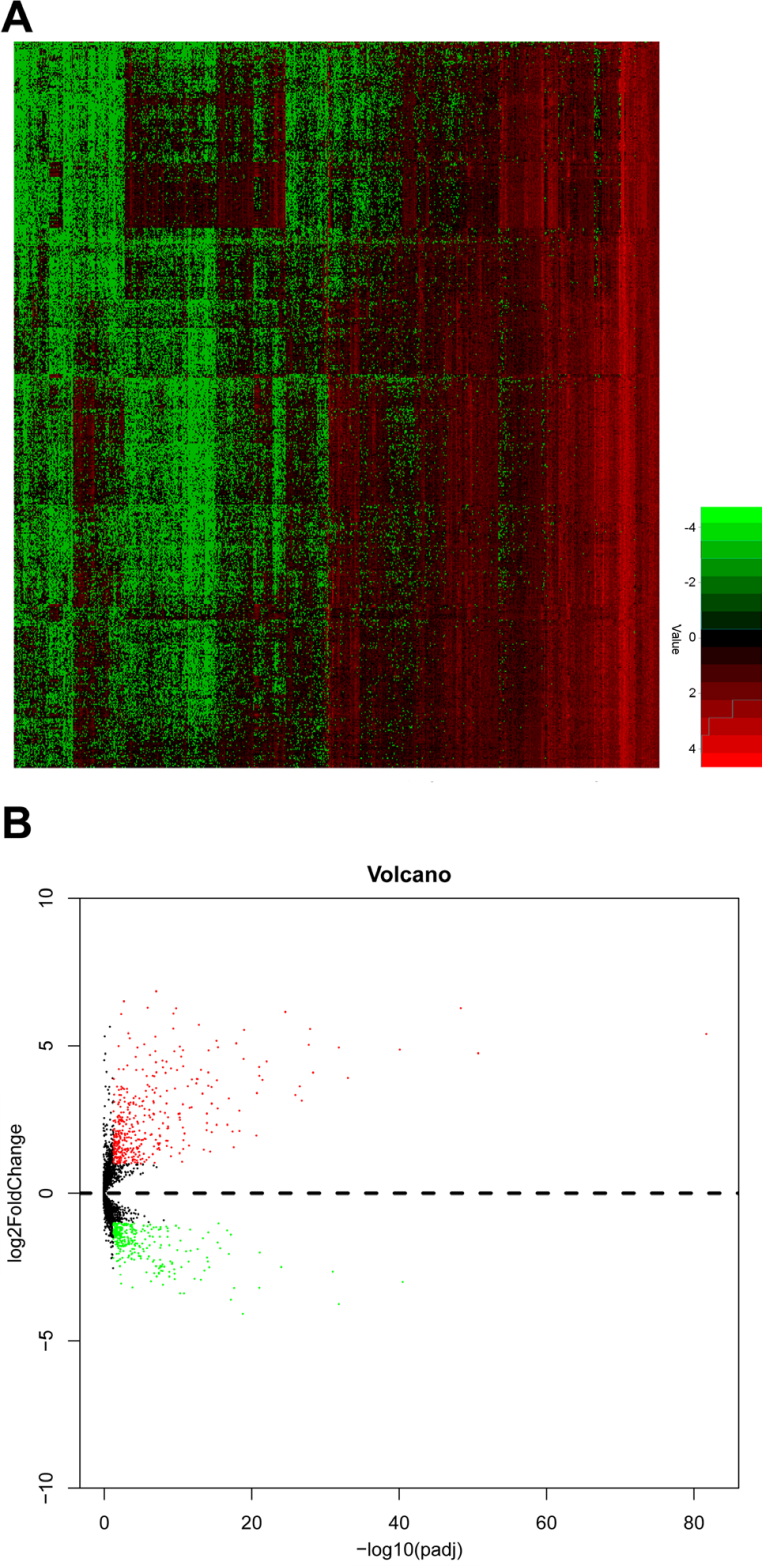
The expression level of upregulated and downregulated lncRNAs in PTC tissues (**A**) heat map; (**B**) volcano map.

Initially, detailed information of 507 PTC patients and 59 normal thyroid tissues was obtained from TCGA dataset. A sum of 734 lncRNAs were selected as differentially expressed lncRNA in PTC compared to normal thyroid tissues, of which 309 lncRNAs were down-regulated and 425 lncRNAs were up-regulated. The overview of aberrant expression lncRNAs was shown in Figure 1A, 1B.

### Specific-lncRNAs correlated with clinical parameters and overall survival (OS)

**Figure 2:**
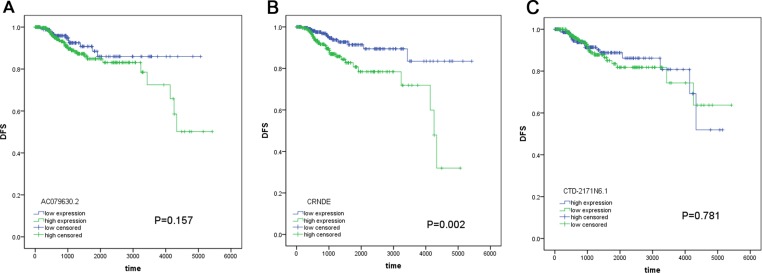
The prognostic significance of these lncRNAs for DFS (**A**) AC079630.2; (**B**) CRNDE; (**C**) CTD-2171N6.1.

With respect to the correlation between lncRNAs and OS, 33 lncRNAs were assessed to be significantly associated with OS of PTC patients by univariate analysis (Table 1). In the light of multivariate analysis, a total of 3 lncRNAs (AC079630.2, CRNDE and CTD-2171N6.1) were remarkably correlated with OS and these lncRNAs were subjected to further analysis (Table 2). We also investigated the association between these lncRNAs and disease-free survival (DFS), but limited predicted significance was observed (Figure 2). Fifty-three patients were excluded for the reason that they lacked specific lncRNAs (AC079630.2, CRNDE or CTD-2171N6.1) expression data. Eventually, 449 patients were included for further analysis in prognosis model. The available data of clinicopathological parameters of the involved patients were summarized in Table 3. Spearman rank correlation was performed to determine the association between these three lncRNAs and clinicopathological variables (Table 4). The results indicated that AC079630.2 was prominently related to tumor size (*P* = 0.005), CRNDE was remarkably related to gender (*P* = 0.016) and CTD-2171N6.1 was significantly related to tumor subtypes (*P* = 0.001). Additionally, the three lncRNAs-based risk score was observed to be notably related to age (*P* = 0.004) and tumor subtypes (*P* = 0.013).

**Table 1:** Specific lncRNAs significantly associated with OS by univariate analysis (TCGA dataset, *N* = 502)

LncRNAs name	HR	*P*
AC079630.2	0.818	0.002
CRNDE	1.796	0.003
CTD-2171N6.1	1.526	0.009
RP3-449M8.9	0.647	0.002
KB-208E9.1	0.670	0.004
RP11-320N7.2	0.707	0.004
RP11-476D10.1	0.827	0.005
LINC00900	0.613	0.005
RP11-1134I14.8	0.685	0.005
RP4-665J23.1	1.643	0.009
KB-1732A1.1	1.946	0.011
RP11-20J15.3	0.815	0.011
RPL34-AS1	1.898	0.012
CITF22-49E9.3	1.658	0.014
RP11-359G22.2	0.727	0.018
RP11-539L10.2	0.726	0.022
AP001258.4	0.591	0.024
MIR100HG	1.539	0.029
RP11-324E6.10	0.626	0.030
RP11-193M21.1	1.601	0.030
RP11-120D5.1	1.822	0.030
CTC-523E23.1	0.646	0.030
RP11-539G18.3	1.549	0.030
RP11-879F14.2	0.678	0.031
RP11-221N13.3	0.827	0.032
RP11-295M3.4	0.630	0.033
AC104655.3	0.765	0.034
RP11-299H21.1	1.589	0.044
LINC01314	0.829	0.045
RP11-42O15.3	1.532	0.046
CH17-373J23.1	1.602	0.046
RP3-449M8.6	0.767	0.047
LINC00284	0.801	0.049

HR: hazard ratio.

**Table 2:** The three lncRNAs significantly correlated with OS by univariate and multivariate analysis in PTC patients (TCGA dataset, *N* = 502)

LncRNAs name	Ensemble ID	Location	Univariate analysis		Multivariate analysis	stepwise	regression
			HR	*P*	β	HR (95% CI)	*P*
AC079630.2	ENSG00000223914	Chromosome 12: 40,156,239–40,167,707	0.818	0.002	−0.344	0.709 (0.548–0.918)	0.009
CRNDE	ENSG00000245694	Chromosome 16: 54,845,189–54,929,189	1.796	0.003	0.787	2.196 (1.044–4.622)	0.038
CTD-2171N6.1/LINC01929	ENSG00000267013	Chromosome 18: 55,105,904–55,124,306	1.526	0.009	0.885	2.422 (1.357–4.321)	0.003

**Table 3:** The characteristics of included PTC patients (TCGA dataset, *N* = 449)

Pathological parameters		No. of patients *N* (%)
Age	< 45	198 (45.3)
	≥ 45	239 (54.7)
Gender	Male	122 (27.9)
	Female	315 (72.1)
Tumor subtypes	Classical/Usual	310 (70.9)
	Tall cell	33 (7.6)
	Follicular	89 (20.4)
	Others	5 (1.1)
History of other tumor	No	406 (94.2)
	Yes	25 (5.8)
Pathological stage	I–II	285 (65.5)
	III–IV	150 (34.5)
T stage	T1–T2	264 (60.4)
	T3–T4	173 (39.6)
N stage	Nx–N0	236 (54.0)
	N1	201 (46.0)
M stage	M0	428 (98.2)
	M1	8 (1.8)
Focus types	Unifocal	228 (53.3)
	Multifocal	200 (46.7)
Extrathyroidal extension	No	280 (66.2)
	Yes	143 (33.8)
BRAF^V600E^	Mutation type	212 (48.5)
	Wild type	225 (51.5)

**Table 4:** The correlation of three lncRNAs and risk score with clinicopathological parameters (TCGA dataset, *N* = 449)

Clinicopathological parameters	AC079630.2 (*P*)	CRNDE (*P*)	CTD-2171N6.1 (*P*)	Risk score (*P*)
Age	0.122	0.115	0.092	**0.004**
Gender	0.602	**0.016**	0.077	0.963
Tumor subtypes	0.508	0.226	**0.001**	**0.013**
History of other tumor	0.71	0.265	0.237	0.226
Pathological stage	0.598	0.074	0.523	0.319
T stage	0.612	0.346	0.342	0.303
N stage	0.057	0.698	0.062	0.308
M stage	0.805	0.209	0.137	0.173
Focus types	0.261	0.922	0.393	0.971
Extrathyroidal extension	0.666	0.251	0.264	0.872
BRAF^V600E^	0.982	0.105	**0.045**	**0.006**

*P: P-* values of Spearman rank correlation.

### Specific lncRNAs featured with diagnostic significance

**Figure 3:**
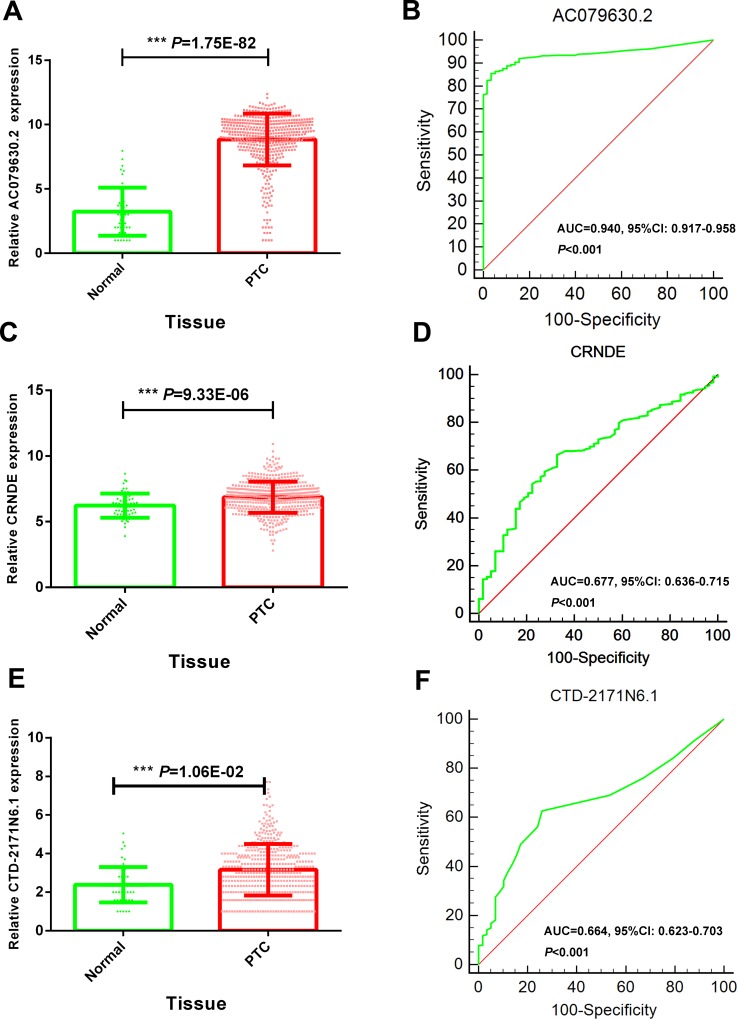
Diagnostic value of 3 novel lncRNAs to distinguish PTC and normal tissues (**A**) Scatter diagram of AC079630.2; (**B**) ROC curve of AC079630.2; (**C**) Scatter diagram of CRNDE; (**D**) ROC curve of CRNDE; (**E**) Scatter diagram of CTD-2171N6.1;(**F**) ROC curve of CTD-2171N6.1.

**Figure 4:**
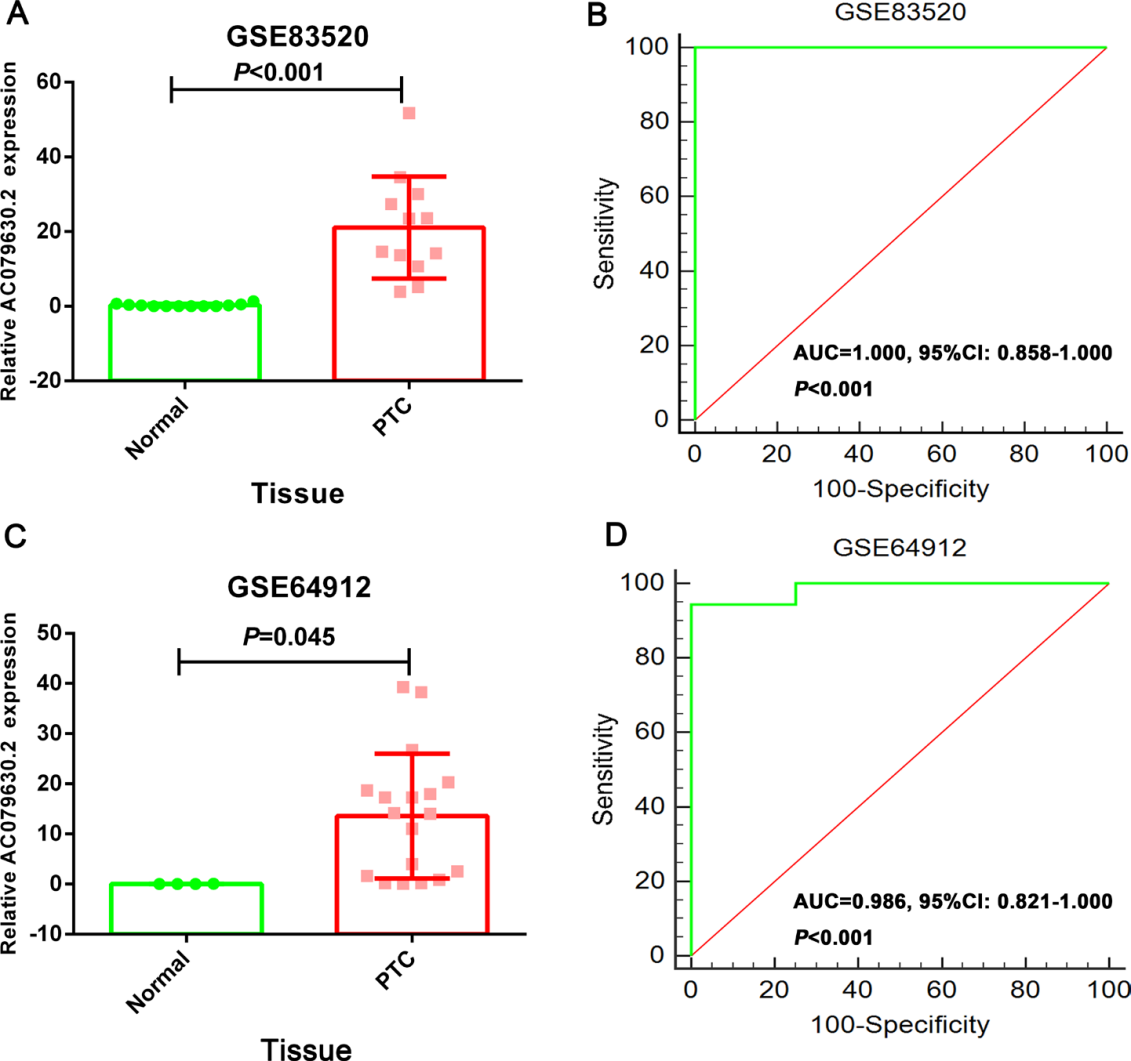
The diagnostic value of AC079630.2 to distinguish PTC and normal tissues (**A**) Scatter diagram of GSE64912; (**B**) ROC curve of GSE64912; (**C**) Scatter diagram of GSE83520; (**D**) ROC curve of GSE83520.

**Figure 5:**
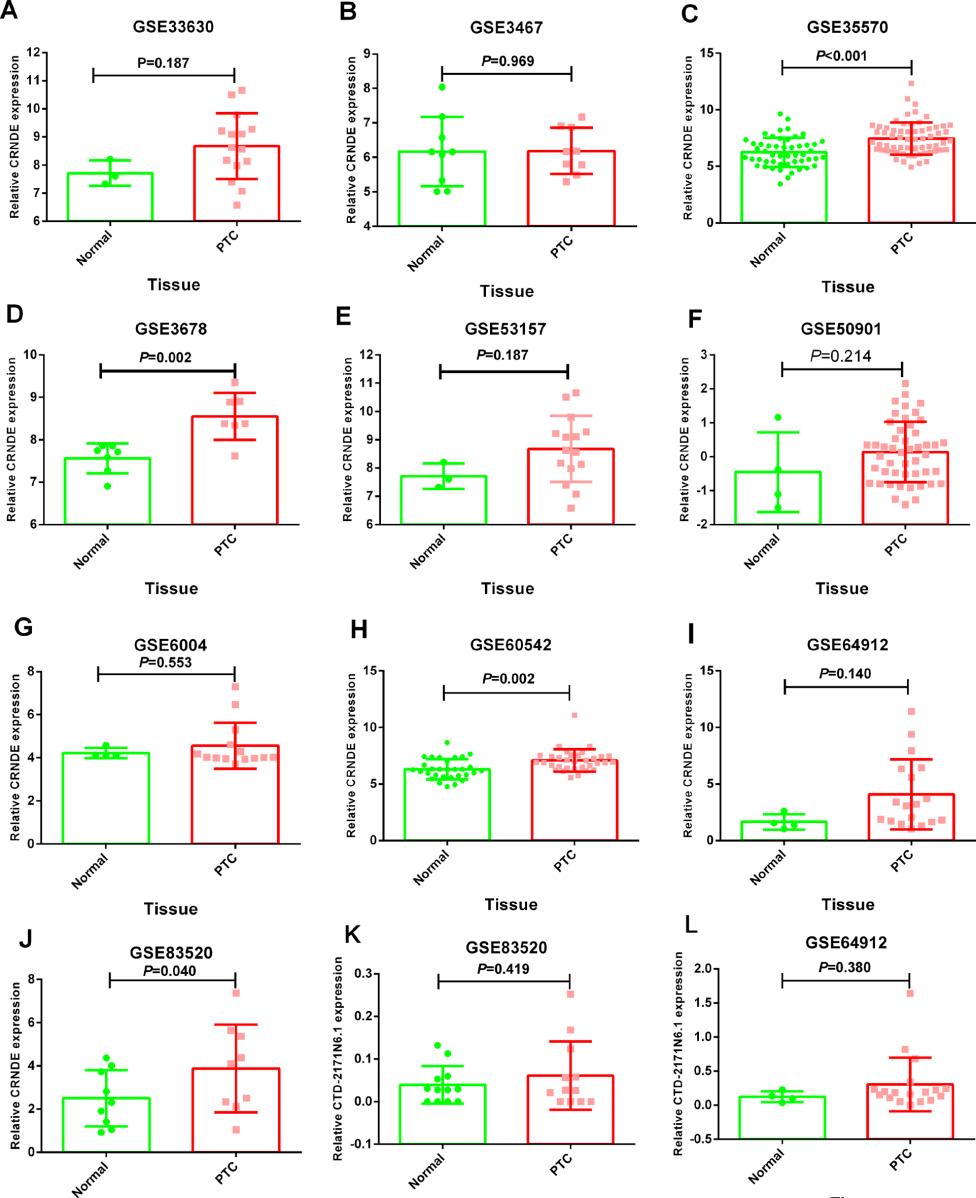
Differential expression of CRNDE and CTD-2171N6.1 in PTC tissues compared to normal tissues (**A**) CRNDE: GSE33630; (**B**) CRNDE: GSE3467; (**C**) CRNDE: GSE35570; (**D**) CRNDE: GSE3678; (**E**) CRNDE: GSE53157; (**F**) CRNDE: GSE50901; (**G**) CRNDE: GSE6004; (**H**) CRNDE: GSE60542; (**I**) CRNDE: GSE64912; (**J**) CRNDE: GSE83520; (**K**) CTD-2171N6.1: GSE83520 (**L**) CTD-2171N6.1: GSE64912.

**Figure 6:**
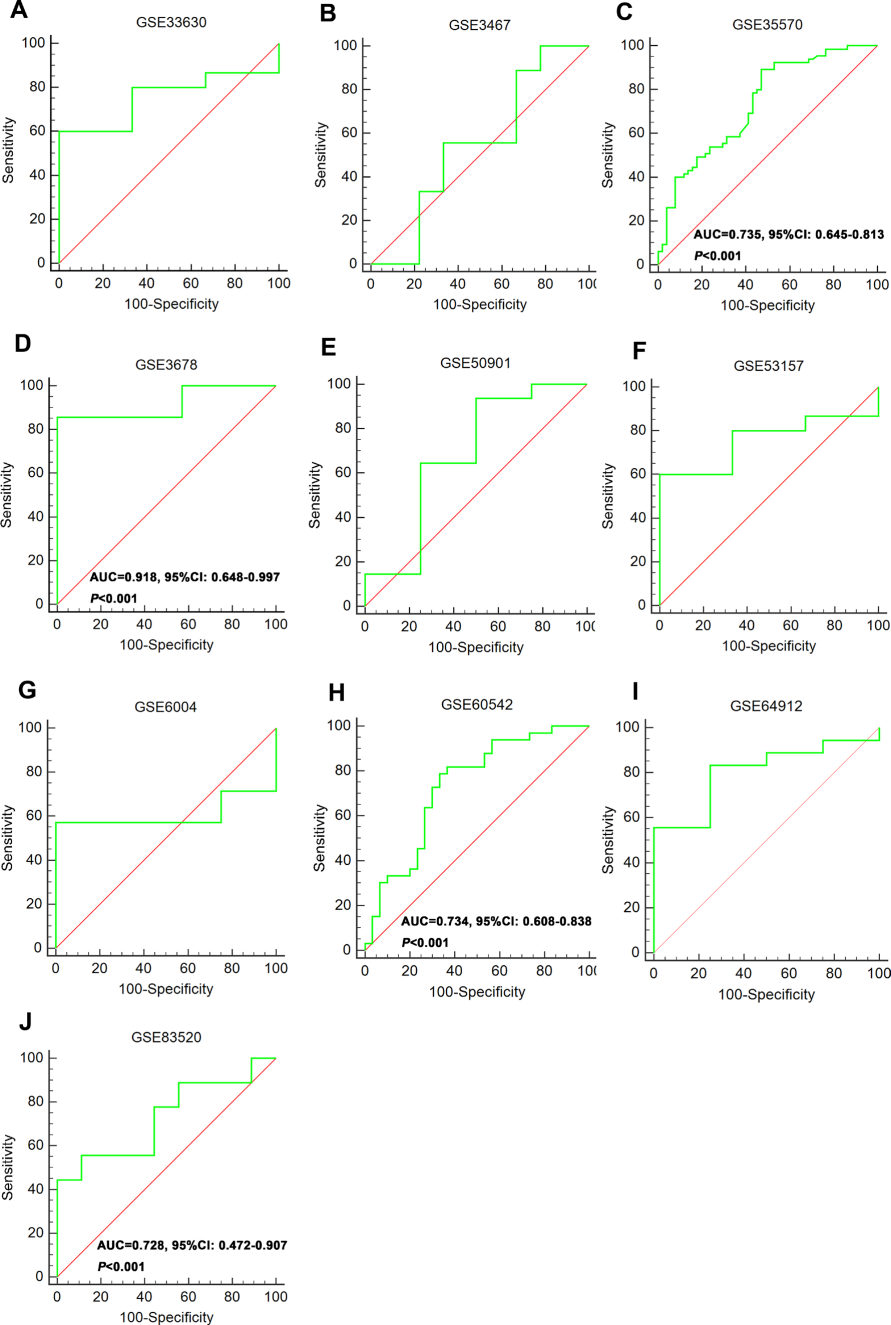
ROC curve of CRNDE to distinguish PTC and normal tissues (**A**) GSE33630; (**B**) GSE3467; (**C**) GSE35570; (**D**) GSE3678; (**E**) GSE50901; (**F**) GSE53157; (**G**) GSE6004; (**H**) GSE60542; (**I**) GSE64912; (**J**) GSE83520.

**Figure 7:**
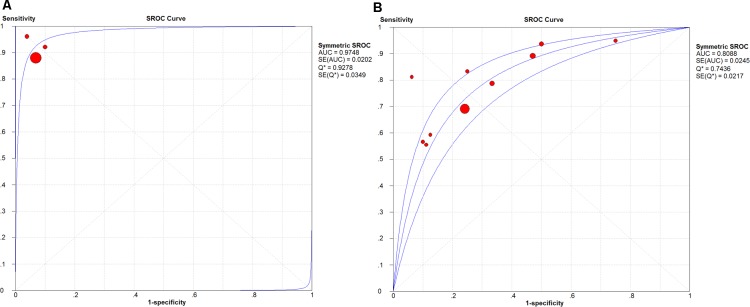
The SROC curve of AC079630 .2 and CRNDE (**A**) AC079630.2; (**B**) CRNDE.

The expression of these three lncRNAs was greatly higher in PTC tissues than that in normal thyroid tissues based on the data from TCGA. Receiver operating characteristic (ROC) curve was conducted to verify the diagnostic value of the three novel lncRNAs in PTC patients. The results of ROC analysis indicated that AC079630.2 exhibited high diagnostic ability to distinguish normal tissue and PTC tissue. The areas under curves (AUC) of these three lncRNAs (AC079630.2, CRNDE and CTD-2171N6.1) were 0.940 (95% CI: 0.917–0.958), 0.677 (95% CI: 0.636–0.715), 0.664 (95% CI: 0.623–0.703), respectively (Figure 3). Moreover, a total of 10 microarrays from GEO were achieved in our study to heighten our finding from TCGA, of which two microarrays contained the data of AC079630.2, 10 for CRNDE, and two for CTD-2171N6.1. The expression level of these three lncRNAs was shown in Table 5. The expression level of AC079630.2 in PTC was evidently higher than that in normal thyroid tissues as shown in both GSE83520 and GSE64912. The AUCs were 1.000 (95% CI: 0.858–1.000) and 0.986 (95% CI: 0.821–1.000), which demonstrated favorable diagnostic value of AC079630.2 in PTC (Figure 4). Among the 10 microarray data containing CRNDE expression, four indicated that CRNDE possessed a certain degree of diagnostic value for PTC, including GSE35570: 0.735 (95%CI: 0.645–0.813), GSE3678: 0.918 (95% CI: 0.648–0.997), GSE60542: 0.734 (95% CI: 0.608–0.838), and GSE83520: 0.728 (95% CI: 0.472–0.907) (Figure 5A–5J and Figure 6). But no significant distinction of CTD-2171N6.1 expression between PTC and normal thyroid was observed in GEO datasets (Figure 5K, 5L). In addition, we conducted meta-analyses of AC079630.2 and CRNDE to explore their diagnostic significances. The results indicated that the pooled AUCs were 0.975 and 0.809 for these two lncRNAs respectively (Figure 7).

**Table 5:** The expression data of 3 novel lncRNAs in GEO datasets

GEO datasets	Year	Country	Platform	Samples	*N*	Relative expression of lncRNAs (mean ± SEM)					
						**AC079630.2**	***P***	**CRNDE**	***P***	**CTD-2171N6.1**	***P***
GSE33630	2012	Belgium	GPL570	PTC	15			8.679 ± 0.3023	0.187		
				Non-cancer	3			7.714 ± 0.2610			
GSE3467	2005	USA	GPL570	PTC	9			6.187 ± 0.2225	0.969		
				Non-cancer	9			6.171 ± 0.3353			
GSE35570	2015	Poland	GPL570	PTC	65			7.464 ± 0.1764	< 0.001		
				Non-cancer	51			6.248 ± 0.1801			
GSE3678	2006	USA	GPL570	PTC	7			8.550 ± 0.2097	0.002		
				Non-cancer	7			7.563 ± 0.1329			
GSE50901	2014	Brazil	GPL13607	PTC	48			0.141 ± 0.1281	0.214		
				Non-cancer	4			−0.454 ± 0.5862			
GSE53157	2013	Portugal	GPL570	PTC	15			8.679 ± 0.3023	0.187		
				Non-cancer	3			7.714 ± 0.2610			
GSE6004	2006	USA	GPL570	PTC	14			4.557 ± 0.2859	0.553		
				Non-cancer	4			4.224 ± 0.1183			
GSE60542	2015	Belgium	GPL570	PTC	33			7.089 ± 0.1708	0.002		
				Non-cancer	30			6.322 ± 0.1642			
GSE64912	2015	Italy	GPL11154	PTC	18	13.580 ± 2.9320	0.045	4.079 ± 0.7280	0.140	0.304 ± 0.0926	0.380
				Non-cancer	4	0.040 ± 0.0209		1.651 ± 0.3393		0.124 ± 0.0400	
GSE83520	2016	USA	GPL16791	PTC	12	21.090 ± 3.9450	< 0.001	3.895 ± 0.5877	0.040	0.061 ± 0.0232	0.419
				Non-cancer	12	0.290 ± 0.1210		2.395 ± 0.3550		0.039 ± 0.0127	

### Prognostic significance of clinicopathological parameters and the three-lncRNA signature in PTC

**Figure 8:**
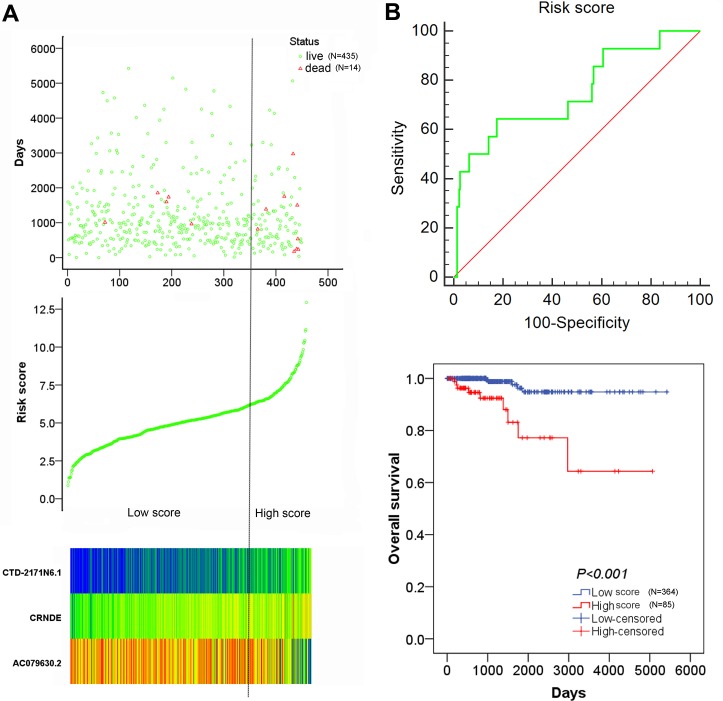
The risk score analysis of 449 PTC patients (**A**) top: survival status of PTC patients; middle: risk score of lncRNAs signature; bottom: risk score of 3 lncRNAs; (**B**) top: the diagnostic significance of risk score to survival outcome; bottom: the association of risk score with OS.

For each patient, a specific risk score was calculated with 3 recruited lncRNAs (AC079630.2, CRNDE and CTD-2171N6.1). The risk score = −0.344 × level of AC079630.2 + 0.787 × level of CRNDE + 0.885 × level of CTD-2171N6.1. The coefficients above were obtained from the β value in Table 2. Then, all patients were divided into low risk group (*N* = 364) and high risk group (*N* = 85) by optimal cut off value. The expression value of the 3 lncRNAs was performed in the heatmap (Figure 8A). The risky lncRNAs (CRNDE, CTD-2171N6.1) feature high expression in high risk group, while the protective lncRNA (AC079630.2) expresses decreased level in high risk group. The Kaplan–Meier survival curve indicated that patients with high risk score were significantly associated with poor prognosis in PTC (Figure 8B). Considering the correlation of risk score with clinicopathological features, the results of Spearman rank correlation revealed that 3 lncRNAs-based risk score was notably related to age (*P* = 0.004) and tumor subtypes (*P* = 0.013), which indicating that elder patients had a higher risk score.

**Figure 9:**
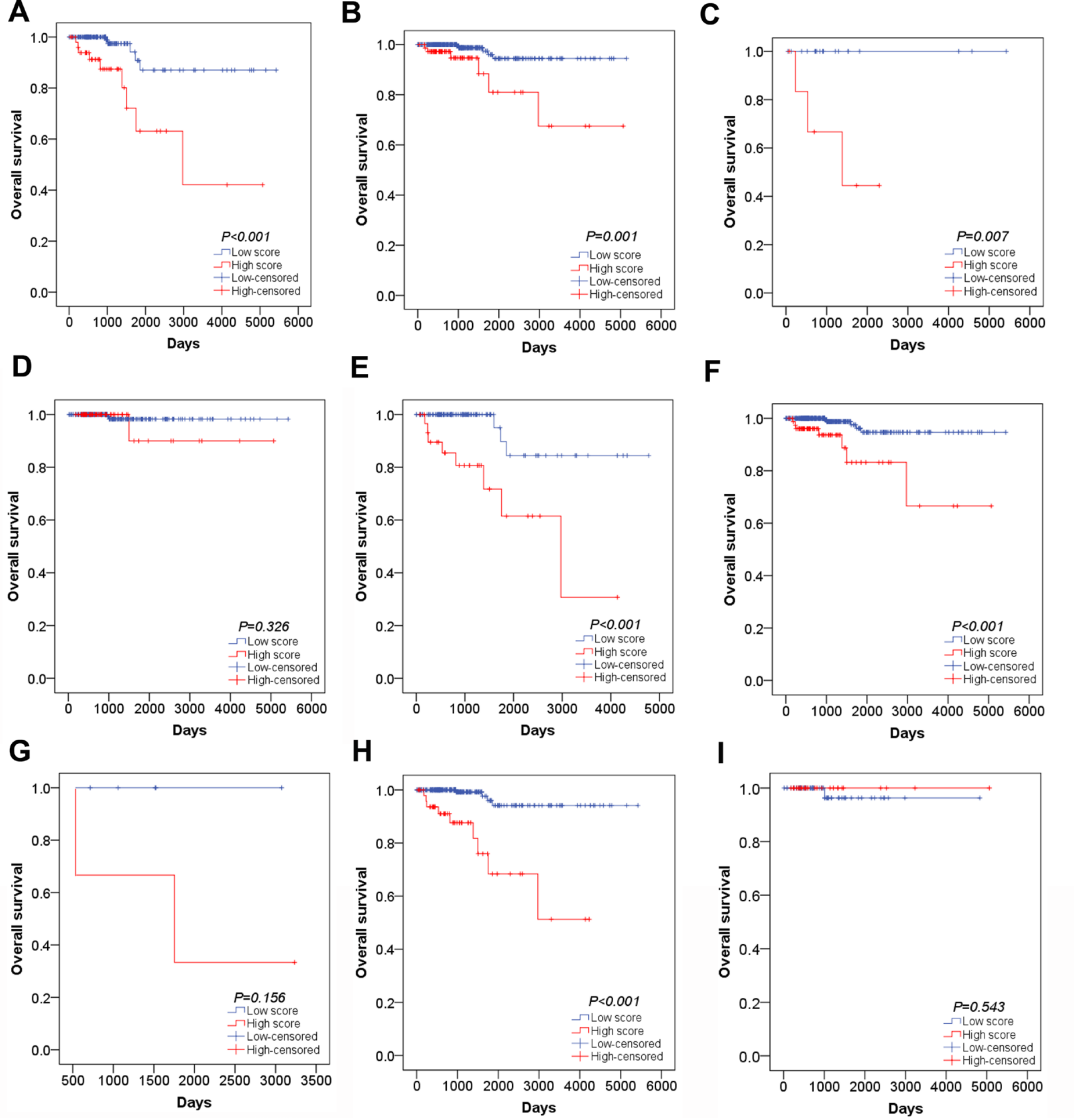
Subgroup analysis of the association between risk score and survival outcome (**A**) Kaplan–Meier survival curve in elder patients; (**B**) Kaplan–Meier survival curve in patients without the other tumor; (**C**) Kaplan–Meier survival curve in patients with the other tumor; (**D**) Kaplan–Meier survival curve in early stage patients; (**E**) Kaplan–Meier survival curve in advanced stage patients;(**F**) Kaplan–Meier survival curve in M0 stage patients; (**G**) Kaplan–Meier survival curve in M1 stage patients; (**H**) Kaplan–Meier survival curve in classical/usual PTC patients; (**I**) Kaplan–Meier survival curve in follicular PTC patients.

The univariate and multivariate Cox proportional hazards regression tests were carried out to explore the prognostic value of clinical factors and risk score. For univariate analysis, we found that age, history of other tumor, pathological stage, M stage and risk score were remarkably associated with worse survival status (*P* < 0.05). After adjusting concomitant variables, the results of multivariate analysis indicated that age (HR = 1.148, 95% CI: 1.070–1.232, *P* < 0.001) and risk score were independent prognostic indicators in PTC (HR = 5.650, 95% CI: 1.188–26.875, *P* = 0.030) (Table 6). In addition, Kaplan–Meier survival curve and log-rank method were further used for the subgroup of clinical features which were significantly associated with OS in univariate analysis. For younger patients, survival analysis was not conducted as all patients were censored. The OS of patients with high risk score were remarkably lower than those with low risk score in elder patients (Figure 9A, *P* < 0.001). When patients were divided into two groups of with and without the history of other tumors, the risk score model exhibited significant prognostic power in both two subgroups (Figure 9B, *P* = 0.007; Figure 9C, *P* = 0.001). Similarly, survival analysis was performed for subgroups of early stage (I–II) and advanced stage (III–IV). The results indicated that the OS time of high risk score group were significant shorter than that of low risk score in patients with advanced stage (Figure 9E, *P* < 0.001). However, no clear difference was observed in patients with early stage (Figure 9D, *P* = 0.326). Furthermore, significant difference of OS time was detected between high risk score group and low risk score group in patients with M0 stage (Figure 9F, *P* < 0.001) but not M1 stage (Figure 9G, *P* = 0.156). In subtypes of tall cell PTC, survival analysis failed to be performed because of lacking ending events. Interestingly, the OS time of classical/usual PTC patients with low risk score was significantly longer than those with high risk score (Figure 9H, *P* < 0.001) and there was no statistical significance among follicular PTC patients (Figure 9I, *P* = 0.0543). Unfortunately, no survival data of AC079630.2, CRNDE or CTD-2171N6.1 in PTC could be obtained from GEO datasets.

**Table 6:** The univariate and multivariate analysis of clinicopathological parameters and prognostic model for OS in PTC patients (TCGA dataset, *N* = 449)

Groups	Univariate analysis		Multivariate analysis	
	**HR (95% CI)**	***P***	**HR (95% CI)**	***P***
Age^a^	1.121 (1.076–1.167)	**< 0.001**	1.149 (1.074–1.229)	**< 0.001**
Gender	0.791 (0.247–2.531)	0.692	1.647 (0.239–11.352)	0.613
Tumor subtypes	0.489 (0.165–1.452)	0.198	0.306 (0.070–1.339)	0.116
History of other tumor	4.163 (1.158–14.971)	**0.029**	2.751 (0.370–20.445)	0.323
Pathological stage	2.683 (1.585–4.540)	**< 0.001**	1.523 (0.491–4.723)	0.466
T stage	3.153 (0.985–10.089)	0.053	0.745 (0.031–18.065)	0.856
N stage	1.421 (0.492–4.103)	0.516	0.456 (0.087–2.399)	0.354
M stage	5.258 (1.164–23.755)	**0.031**	0.748 (0.048–11.568)	0.835
Focus types	0.283 (0.063–1.273)	0.100	0.115 (0.009–1.414)	0.091
Extrathyroidal extension	2.660 (0.885–7.992)	0.081	0.757 (0.049–11.676)	0.842
BRAF^V600E^	0.830 (0.274–2.517)	0.743	1.664 (0.321–8.633)	0.544
Risk score	8.592 (2.878–25.651)	**< 0.001**	7.092 (1.667–30.179)	**0.008**

HR: hazard ratio; CI: confidence interval; ^a^continuous.

### Functional and protein-protein interaction (PPI) analysis of correlative genes of these lncRNAs

**Figure 10:**
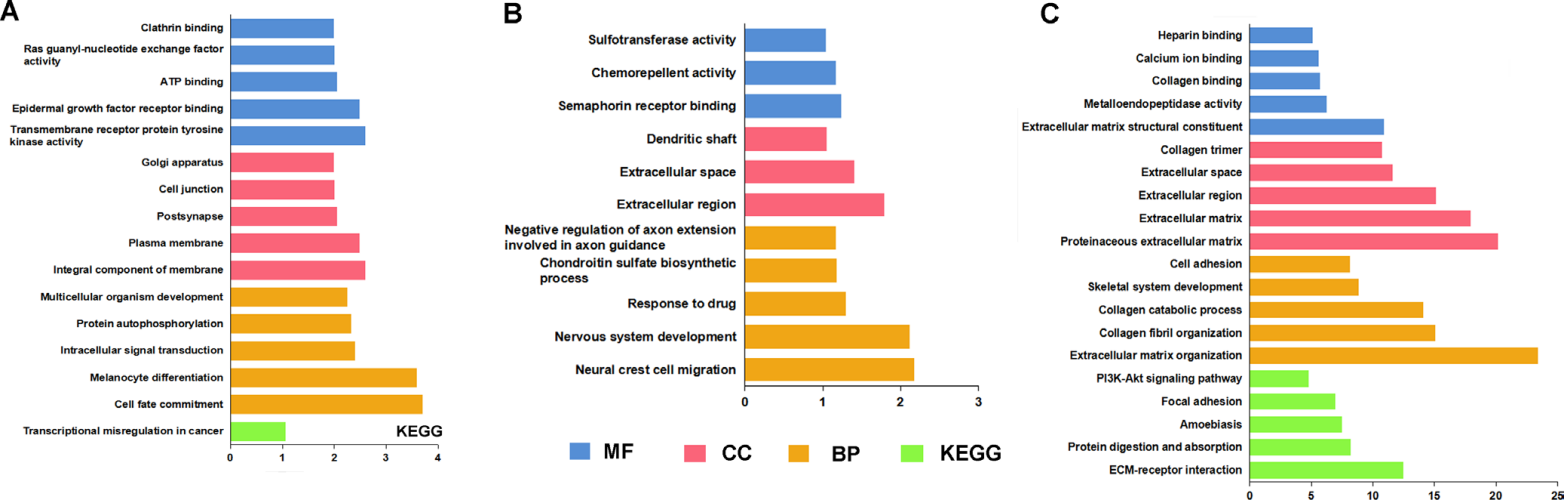
GO terms and KEGG pathways analysis by target genes of 3 lncRNAs (**A**) AC079630.2; (**B**) CRNDE; (**C**) CTD-2171N6.1.

**Figure 11:**
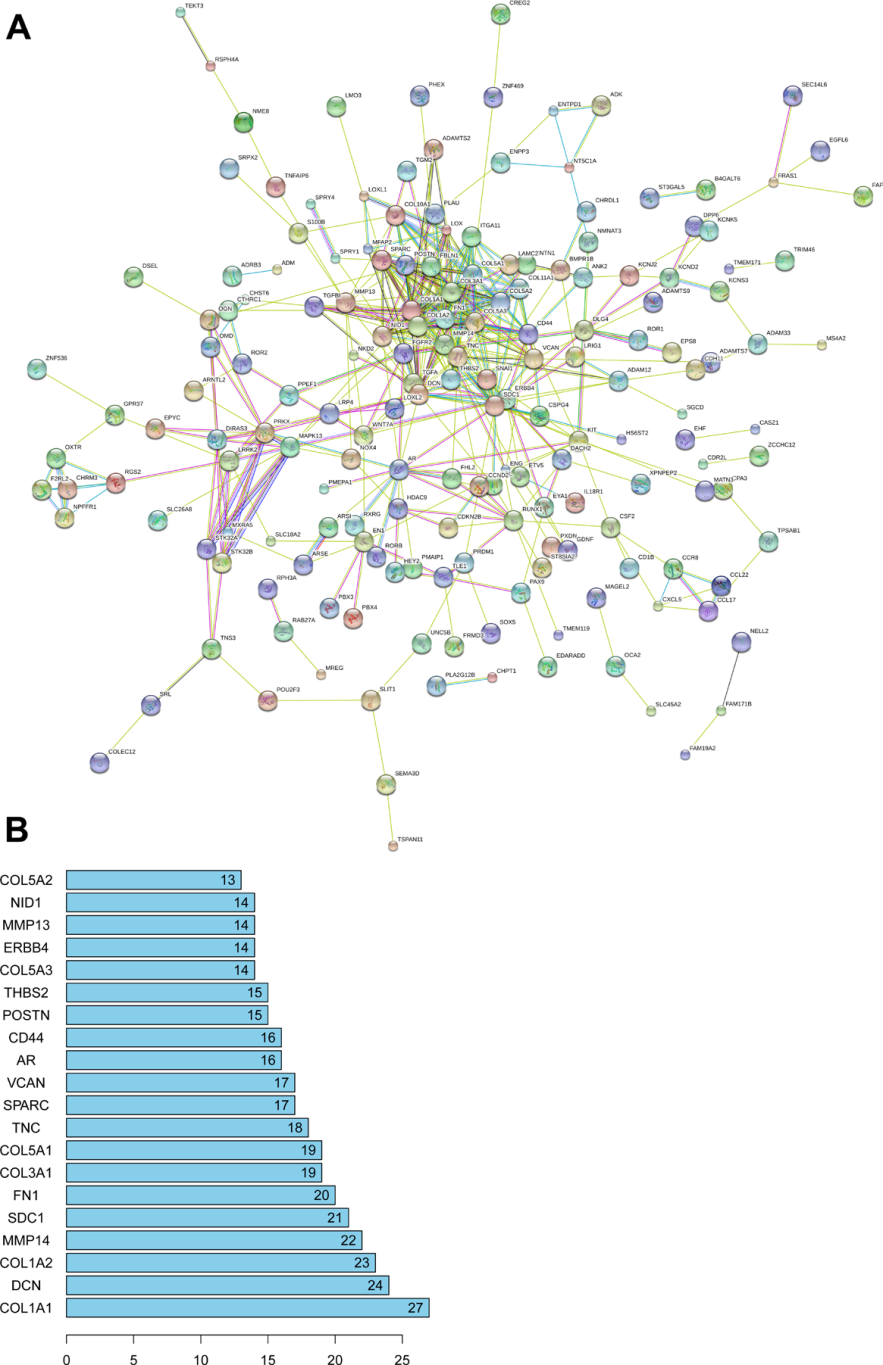
PPI network of 408 target genes of 3 novel lncRNAs (**A**) map of PPI network; (**B**) core genes of PPI network.

A total of 4086 genes were dysregulated in PTC tissues, of which 409 were identified as lncRNA-correlative genes. All the statistically significant results of functional and pathway enriched analysis were shown in Table 7. As for Gene Ontology (GO) terms, the top 5 significantly enriched terms were listed in Table 7. The results of Kyoto Encyclopedia of Genes and Genomes (KEGG) analysis indicated that the related genes of AC079630.2 were mostly enriched in the pathway of “Transcriptional misregulation in cancer” and the related genes of CTD-2171N6.1 were mostly enriched in the pathways of “ECM-receptor interaction”, “Protein digestion and absorption”, “Amoebiasis”, “Focal adhesion” and “PI3K-Akt signaling pathway” (Figure 10A–10C). However, there was no pathway significantly enriched by the related genes of CRNDE as assessed by the pathway analyses above. The PPI network was showed in Figure 11. Moreover, with the rank of degree, the top 20 genes (COL1A1, DCN, COL1A2, MMP14, SDC1, FN1, COL3A1, COL5A1, TNC, SPARC, VCAN, AR, CD44, POSTN, THBS2, COL5A3, ERBB4, MMP13, NID1 and COL5A2) were identified as core genes which participate in the progression of PTC.

**Table 7:** GO and KEGG enriched analysis for lncRNAs-related genes

Enriched analysis	Targets count	*P* value	FDR
**AC079630.2**			
**GO terms (BP)**			
GO:0045165∼cell fate commitment	5	2.00E-04	3.09E-01
GO:0030318∼melanocyte differentiation	4	2.58E-04	3.99E-01
GO:0035556∼intracellular signal transduction	9	4.00E-03	6.03E+00
GO:0046777∼protein autophosphorylation	6	4.69E-03	7.03E+00
GO:0007275∼multicellular organism development	10	5.70E-03	8.48E+00
**GO terms (CC)**			
GO:0016021∼integral component of membrane	49	2.56E-03	3.09E+00
GO:0005886∼plasma membrane	41	3.28E-03	3.95E+00
GO:0098794∼postsynapse	3	8.82E-03	1.03E+01
GO:0030054∼cell junction	9	1.00E-02	1.16E+01
GO:0005794∼Golgi apparatus	13	1.01E-02	1.17E+01
**GO terms (MF)**			
GO:0004714∼transmembrane receptor protein tyrosine kinase activity	4	1.97E-03	2.51E+00
GO:0005154∼epidermal growth factor receptor binding	3	1.76E-02	2.04E+01
GO:0005524∼ATP binding	17	3.52E-02	3.69E+01
GO:0005088∼Ras guanyl-nucleotide exchange factor activity	4	4.02E-02	4.10E+01
GO:0030276∼clathrin binding	3	4.59E-02	4.53E+01
**KEGG analysis**			
hsa05202:Transcriptional misregulation in cancer	4	8.60E-02	6.40E+01
**CRNDE**			
**GO terms (BP)**			
GO:0001755∼neural crest cell migration	3	6.74E-03	9.02E+00
GO:0007399∼nervous system development	5	7.76E-03	1.03E+01
GO:0042493∼response to drug	4	5.04E-02	5.14E+01
GO:0030206∼chondroitin sulfate biosynthetic process	2	6.63E-02	6.16E+01
GO:0048843∼negative regulation of axon extension involved in axon guidance	2	6.89E-02	6.31E+01
**GO terms (CC)**			
GO:0005576∼extracellular region	11	1.64E-02	1.61E+01
GO:0005615∼extracellular space	9	3.96E-02	3.49E+01
GO:0043198∼dendritic shaft	2	8.91E-02	6.29E+01
**GO terms (MF)**			
GO:0030215∼semaphorin receptor binding	2	5.83E-02	5.05E+01
GO:0045499∼chemorepellent activity	2	6.81E-02	5.62E+01
GO:0008146∼sulfotransferase activity	2	9.21E-02	6.78E+01
**CTD-2171N6.1**			
**GO terms (BP)**			
GO:0030198∼extracellular matrix organization	25	4.16E-24	6.26E-21
GO:0030199∼collagen fibril organization	12	8.10E-16	1.17E-12
GO:0030574∼collagen catabolic process	13	8.37E-15	1.25E-11
GO:0001501∼skeletal system development	12	1.52E-09	2.29E-06
GO:0007155∼cell adhesion	18	7.37E-09	1.11E-05
**GO terms (CC)**			
GO:0005578∼proteinaceous extracellular matrix	25	7.15E-21	8.27E-18
GO:0031012∼extracellular matrix	24	1.23E-18	1.42E-15
GO:0005576∼extracellular region	43	7.28E-16	8.99E-13
GO:0005615∼extracellular space	35	2.50E-12	2.89E-09
GO:0005581∼collagen trimer	12	1.81E-11	2.09E-08
**GO terms (MF)**			
GO:0005201∼extracellular matrix structural constituent	11	1.25E-11	1.55E-08
GO:0004222∼metalloendopeptidase activity	9	5.90E-07	7.33E-04
GO:0005518∼collagen binding	7	2.14E-06	2.65E-03
GO:0005509∼calcium ion binding	18	2.55E-06	3.17E-03
GO:0008201∼heparin binding	9	8.05E-06	9.99E-03
**KEGG analysis**			
hsa04512:ECM-receptor interaction	13	3.51E-13	3.70E-10
hsa04974:Protein digestion and absorption	10	6.50E-09	6.85E-06
hsa05146:Amoebiasis	10	3.40E-08	3.59E-05
hsa04510:Focal adhesion	12	1.09E-07	1.15E-04
hsa04151:PI3K-Akt signaling pathway	12	1.75E-05	1.85E-02

GO: Gene Ontology; KEGG: Kyoto Encyclopedia of Genes and Genomes; BP biological process; CC cellular component; MF molecular function.

## DISCUSSION

In this study, the genome-wide high-throughput RNA-sequencing data of lncRNA expression level of PTC were retrieved from TCGA dataset. Survival analysis revealed that three lncRNAs (AC079630.2, CRNDE and CTD-2171N6.1) were pronouncedly related to the patient survival. More importantly, a risk score calculated by the expression level of these three lncRNAs was an independent prognostic factor of PTC.

Recent researches have revealed that lncRNAs play critical roles in cancers, including thyroid cancer [25–27]. The clinical role of a certain lncRNAs has been well documented. For example, remarkably higher expression of HOX transcript antisense RNA (HOTAIR) was detected in PTC tissues versus normal tissues [28].The expression of antisense non-coding RNA in the INK4 locus (ANRIL) was also distinctly up-regulated in thyroid cancer tissues and cells, and closely related to histological grade and status of lymph node metastasis [29]. Similarly, the BRAF-activated non-coding RNA (BANCR) levels were prominently lower in PTC tumor tissues than in control tissues. Decreased BANCR levels were related to tumor size, the presence of multifocal lesions and advanced stage [30]. Furthermore, another lncRNA, PTCSC3, was strongly downregulated in PTC tissues [31]. Not many lncRNAs have been verified to be effective indicators for the progression or prognosis prediction. A functional lncRNA, SLC6A9-5:2, was found to be down-regulated in PTC and it was involved in the radioactive therapy resistance of PTC [32]. However, only clinical role of one single lncRNA was studied in aforementioned studies.

Profiling of lncRNAs based on the techniques of microarray or RNA-sequencing can greatly facilitate the discovery of novel lncRNA candidates in PTC. A genome-wide analysis of lncRNA expression profile with microarray in PTC with 62 cases revealed that 3499 lncRNAs were distinctly expressed. Quantitative real time polymerase chain reaction (qRT-PCR) was employed to verify randomly 10 distinctly expressed lncRNAs, including TCONS_l2_00010365, n386477, n340790, lnc-LLPH-2:1 and 261 NR_003225.2 being up-regulated, and lnc-PSD4-1:14, n335550, lnc-KCMF1-2:1, lnc-PLA2R1-1:1 and ENST00000422494.1 being down-regulated [19]. Another microarray study based on three patients of PTC indicated that 675 lncRNAs were distinctly expressed in PTC samples versus adjacent noncancerous samples. To confirm the microarray results, eight abnormally expressed lncRNAs were also randomly selected for detection with RT-qPCR with 36 cases, including 4 upregulated (ENST00000503723, ENST00000423539, uc003tab.3 and NR_073085) and 4 downregulated (ENST00000515275, ENST00000570022, uc003qef.1 and ENST00000427243) [20]. In addition to microarray, next-generation sequencing (NGS) in thyroid cancer allows concurrent high-throughput sequencing exploration of variable genetic changes and thus can offer a wide-ranging cognition of thyroid cancer biology [33]. RNA sequencing was performed to identify the changes to the transcriptome profile in 12 thyroid tissues compared to paired normal adjacent tissues. A total of 188 lncRNAs were detected to be abnormally expressed in 50% or more of the thyroid tissues as assessed by RNA-sequencing compared with paired normal adjacent tissues. Forty-seven lncRNAs were verified in 31 pairs of PTC specimens using RT-qPCR. The lncRNAs NONHSAT076747 and NONHSAT122730 were found to be related to lymph node metastasis, and NONHSAG051968 expression was adversely related to tumor size [21]. Whole exome sequencing was also performed by another group with 91 pairs of PTC tissues. They further performed Sanger sequencing with 311 pairs of samples. Interestingly, they found that lncRNA GAS8-AS1 is the second most commonly differentiated gene and functions as a new tumor suppressive lncRNA in thyroid [22]. However, most of the studies above were performed based on small number of patients. Due to different technique platforms, distinct setting for cut-off value, various patient sources, the profiling of differentially expressed lncRNAs was inconsistent. Furthermore, the prognostic role of these lncRNAs has not been explored.

In the light of the established molecular aberrations, the TCGA dataset has accomplished a comprehensive genetic and epigenetic investigation into a large cohort of thyroid cases by the most innovative approaches of next-generation sequencing [34–36]. To the best of our knowledge, there has been only one paper so far, which has mined the high-throughput data of lncRNAs of PTC and was published when we were preparing for our paper. Ma et al. [24] reported that 220 lncRNAs were altered expressed from the annotated 2773 lncRNAs which were approved by the HUGO gene nomenclature committee in TCGA dataset. Among these lncRNAs, FAM41C, CTBP1-AS2, LINC00271, HAR1A, LINC00310 and HAS2-AS1 were found to be related to patient recurrence. After the classical clinicopathological factors and BRAF^V600E^ mutation were adjusted, LINC00271 was proved to be an independent risk factor for a series of clinical characteristics, including extrathyroidal extension, lymph node metastasis, advanced tumor stage III/IV and recurrence in multivariate analyses. The results of our study were different with that published by Ma et al. The reasons might be that Ma et al. [24] carried out the expression alteration of the lncRNAs using The cBioPortal for Cancer Genomics, while in the current study, DEseq R package was used, which is widely applied for the analysis of RNA-seq data. Furthermore, we annotated 7589 lncRNAs, more than Ma et al. did, using Ensembl genome browser (http://www.ensembl.org/), which supports the result of our current study. Collectively, based on 507 thyroid patients and 59 normal thyroid tissues, we identified 734 differentially expressed lncRNAs, among which 33 lncRNAs were evaluated to be significantly associated with OS of thyroid patients by univariate analysis and eventually, the prognostic values of three lncRNAs (AC079630.2, CRNDE and CTD-2171N6.1) were confirmed by multivariate analysis. Generally, DFS is more appropriate to assess the outcome of PTC patients. Actually, we also investigated the association of these lncRNAs with DFS, but the results indicated that only CRNDE was significantly associated with DFS. A specific risk score was calculated with these three lncRNAs and this risk score and age were identified as independent prognostic indicators for PTC. In addition, the risk score was positively correlated with age, which indicated that the elder PTC patients had higher risk score. Furthermore, the aberrant expression of AC079630.2 and CRNDE could be confirmed with other PTC samples based on different detecting methods, namely, microarrays, which strengthens our current findings. We also attempted to validate the prognostic value of the three-lncRNA signature in PTC based on GEO datasets. Regrettably, no survival data of AC079630.2, CRNDE or CTD-2171N6.1 in PTC was available in GEO datasets. The genetic alteration of lncRNAs was observed in various tumors and its alteration can assist to predict patients’ survival [37, 38]. Nevertheless, no alteration was observed for CRNDE and no relevant information about another two lncRNAs could be achieved in cBioPortal for Cancer Genomics (http://www.cbioportal.org/). Other clinical experiments need to be carried out to verify the prognostic role of the novel three-lncRNA signature in PTC.

Concerning the biological function and clinical role of these lncRNAs, only CRNDE has been studied in cancers so far. Several studies indicated that CRNDE expression significantly increased in gliomas and was related to the tumor progression, recurrence and survival [39, 40]. CRNDE can promote the malignant progress of glioma by lessening miR-384/PIWIL4/STAT3 Axis, miR-186 or mTOR signaling pathways [41–43]. CRNDE has been reported to be a new serum-based marker for diagnosis and prognosis of colorectal cancer [44], and it can stimulate cell growth and chemoresistance of colorectal cancer cells via correlating with IRX5 or miR-181a-5p-mediated regulation of Wnt/β-catenin signaling [45, 46]. Moreover, CRNDE can promote tumor growth in medulloblastoma [47], hepatic carcinoma [48], gallbladder carcinoma [49], renal cell carcinoma [50] as well as in ovarian carcinoma patients [51] with various prospective molecular mechanisms. However, the clinical value and underlying mechanism of CRNDE in PTC remains largely unveiled and no possible pathway could be suggested by our signaling investigation. More exploration is acquired to understand the mechanism of CRNDE in the future. The pathway of “Transcriptional misregulation in cancer” is closely related to AC079630.2 and 5 pathways are linked to CTD-2171N6.1. Interestingly, published studies indicate that some genes can exert its oncogenic function by activating PI3K-Akt signaling pathway in PTC [52, 53], which can be functioned via CTD-2171N6.1. However, relevant validated research needs to be carried out in the future.

One of the limitations of the current study is that most of the cases in the cohort were censored and there were only 14 end events, which might cause some bias to obtain accurate result. Furthermore, no data on the iodine therapy or other details of therapeutic strategies could be achieved. Finally, validation with FISH or qPCR is still needed afterwards.

To sum up, we have identified three novel lncRNAs (AC079630.2, CRNDE and CTD-2171N6.1) which are markedly related to the survival of PTC. The novelty of the current study lies in the special risk score generated by the three lncRNAs, which is an independent prognostic indicator for PTC. The accurate molecular roles of these three lncRNAs identified warrants further characterization, but our current data propose that they are prospective to play pivotal roles in PTC tumorigenesis and more importantly, these novel lncRNAs are closely related to patients’ survival. These results have far-reaching consequences with respect to molecular prediction PTC.

## MATERIALS AND METHODS

### Patient cohort and selection of candidate lncRNAs

The RNA-sequencing and clinical data of 510 PTC patients and 59 normal thyroid tissues were provided by TCGA data center (https://portal.gdc.cancer.gov/). After choosing the maximum expression value when multiple detections were performed for the same patient, 507 PTC patients and 59 normal thyroid tissues were finally included. The type of surgery and whether these patients received radioactive iodine therapy were unclear and the follow-up data was available for 502 patients. To further analyze the association of genes level with clinical data, the expression of genes were log2 transformed and records were considered as censored when the expression level were 0 or 1 [54]. In addition, the genes were eliminated when the censored data were more than 10%. Dysregulated genes were screened by R software using the package “DEseq” and genes with significant differential expression between PTC tissues and normal thyroid tissues were selected by the standard of log2 fold change (FC) and adjusted *P* value. The genes were noted as up-regulated or down-regulated when logFC>1 or logFC<-1 with adjusted *P* < 0.05, respectively. Subsequently, the expression data of dysregulated lncRNAs and survival data were analyzed with univariate Cox proportional hazards regression model. To explore each lncRNA as an independent indicator for survival, multivariate stepwise regression analysis was utilized for lncRNAs which were significantly associated with OS (*P* < 0.05) in univariate analysis. Finally, the diagnostic and prognostic values of those selected lncRNAs were validated using other cohorts from Gene Expression Omnibus (GEO) databases and a meta-analysis was subsequently conducted via the calculation of summarized ROC (sROC).

### Construction of lncRNA-related prognostic indicator

With the coefficient value of multivariate analysis, an lncRNA-related prognostic model was constructed by calculating a risk score for each subject. The risk score = β of lncRNA1 * level of lncRNA1+β of lncRNA2 * level of lncRNA2+…+β of lncRNAn * level of lncRNAn. Additionally, patients were divided into the groups of high risk score and low risk score with the optimal cut off value. And the risk score was regarded as a biomarker for further analysis. Moreover, multivariate Cox proportional hazards regression was performed to adjust classical clinicopathological parameters, such as pathological stage, *T* stage, *N* stage, *M* stage, focus types, extrathyroidal extension and BRAF^V600E^.

### Functional and protein-protein interaction (PPI) analysis for the correlative genes of lncRNAs

Pearson correlation analysis was performed to explore the correlation between dysregulated genes and each lncRNA in prognostic model. According to the ranked coefficients, the top 1% genes were selected as lncRNA-associated genes for further analysis. Then, GO and KEGG enriched analyses, which are functional annotation analyses by bioinformatics enrichment tools using large gene lists [55], were conducted using the related genes of each lncRNA separately in DAVID website (http://david.abcc.ncifcrf.gov/). PPI was performed within the Interacting Genes/Proteins (STRING) database (http://string-db.org/).

### Statistical analysis

HR and corresponding 95% CI were assessed by univariate/multivariate Cox proportional hazards regression model to explore the prognostic impact of clinicopathological parameters and biomarkers. Kaplan–Meier survival curve and log-rank test were conducted to investigate the association between risk score and OS in PTC patients. In addition, we performed a subgroup analysis with clinicopathological parameters which were correlated with OS and subtypes of PTC. ROC curve was implemented to estimate the optimal cut off value of risk score to predict survival status. Simultaneously, Spearman rank correlation was performed to assess the association between three lncRNAs and clinicopathological parameters. Statistical significance threshold was set at a two-side *P* < 0.05. The analyses above were conducted with R software and SPSS 22.0 (SPSS Inc., Chicago, IL, USA).
